# Reconstructing the intellectual neighbourhood: tracing Ali Qushji’s influence in science

**DOI:** 10.12688/f1000research.162047.1

**Published:** 2025-03-13

**Authors:** Nargis Nurulla-Khoja

**Affiliations:** 1 Cultural Heritage, Silk Road International University, Samarkand, Uzbekistan; 2 Samarkand State University named after Sharof Rashidov, Samarkand, Samarkand Province, Uzbekistan

**Keywords:** Samarkand, Astronomy, Islam, neighbourhood, Ulugbek, Ali Qushji, Copernicus

## Abstract

The article introduces the concept of ‘intellectual neighbourhood,’ a historical-philosophical framework emphasising the collaborative nature of knowledge production across time in Eurasia, where respect for diverse ideas flourished. The study traces the contributions of Ali Qushji and his followers, illustrating how their works emerged from this interconnected intellectual milieu. The analysis further highlights how cosmopolitan cities like Samarkand and Istanbul functioned as conduits for knowledge exchange and acknowledges the influence of key Islamic scholars such as Nasir al-Din Tusi. Ultimately, the article advocates for a deeper appreciation of historical contexts to inform the reconstruction of contemporary intellectual neighbourhoods. It underscores the critical importance of trust and collaboration in navigating today’s complex global landscape.

## Introduction

The present discourse examines the historical landscape of the late Medieval period, a pivotal era characterised by profound transformations and the emergence of significant figures who played crucial roles in shaping modern science. It aims to elucidate the complex interplay of ideas and events that defined the time and the multifaceted nature of the circulation of the Eurasian world. ‘Intellectual neighbourhood’ is a historical-philosophical concept of horizontal Eurasian order, where respect for Others and their knowledge flourishes, together with the communication traditions. Such tradition is based on the understanding that knowledge is never an individual project; we need to have and be raised in an environment that fosters compromises in trade, science and diplomacy, allowing for the adaptation of local contexts to incorporate the influences of others. In the presented article, the concept – intellectual neighbourhood – is used as a methodological framework to trace Ali Qushji (1403-1474, known as Ali ibn Muhammad Qushji) and Nikolaus Copernicus (1473-1543).

Given the historical context, the proactive efforts of the organisers of a series of conferences at Istanbul University focused on Ali Qushji’s heritage serve as a powerful and rare example of how the collective contributions of scholars from diverse historical and geographical backgrounds can enhance learning among distant neighbours. ^1^ While it may seem improbable that those events could restore an effective and adaptive intellectual neighbourhood, striving towards this goal is crucial, particularly today. Building greater trust within local communities and their members is critical. Achieving such a transformative shift necessitates a profound understanding of the historical context — the historical ‘space that existed before’ and the present circumstances. Only with this comprehensive understanding can we begin to adapt and truly grasp the intellectual neighbourhood, which transcends modern-day boundaries. It echoes the historical role of cosmopolitan cities such as Samarkand and Istanbul as bridges between neighbours—a legacy epitomised by the distinguished Ulugbek (d. 1449) and Sultan Mehmed II (d.1481).

The article literature review is primarily based on the historical sources used, most of which are the works of key Islamic scholars in astronomy, such as Nasir ad-Din Tusi (1201-1274), Ulugbek, and Ali Qushji. Tusi established the world’s first scientific astronomical institute and observatory in 1262. Later, in 1403, a nine-year-old boy named Ulugbek and his famous teacher Kazizadeh Rumi visited the observatory. Although the observatory was already in decline, this scientific project impressed Ulugbek and inspired him to create something similar. Tusi’s work, observatory and critical examination of Ptolemaic geometry in the *Tajrid al-itiqad* (‘The Purification of Belief’), ^2^ laid a crucial foundation for later developments in mathematical astronomy. It is a subject that we will explore shortly. It paved the way for notable advancements in mathematical astronomy, including his proposition of sophisticated techniques using compound circles with uniform circular motion. This innovative aspect of Tusi’s work, particularly his techniques, created a springboard for subsequent Central Asian astronomers, especially in discussions surrounding the heliocentric model.

The other source is the New Astronomical Table (in Tajik-Persian as ‘Zijji Sultani’ and in Turkish as ‘Zij-i Jadidi Kuragoniy’) co-authored by Ulugbek and Ali Qushji, which stands out as a milestone in the history of astronomy due to its remarkable precision in star cataloguing. ^3^ Furthermore, Ali Qushji’s work, particularly his critique of Ptolemy’s geocentric model in the Mercury Treatise, marks a turning point in the shift towards empirical inquiry in astronomy, challenging established ideas and opening the door to considering Earth’s rotation. ^4^ The article also highlights Claudius Ptolemy’s Almagest’s lasting impacts on Islamic and European astronomy. ^5^ Ptolemy’s geocentric model, developed in Hellenistic Alexandria, became a dominant scientific framework influencing astronomical thought across cultures. The work points to Basilios Bessarion’s role ^6^ in preserving and translating Islamic astronomical texts into Greek, which significantly enriched European scholarship. His efforts laid the groundwork for Regiomontanus, who further advanced European understanding of celestial mechanics through his Latin translation of the Almagest and his own Epytoma published in 1496. ^7^ The analysis suggests that the network of intellectual neighbours created an environment conducive to Copernicus’s revolution.

The structure of the work that follows the Introduction includes a premise that briefly reviews the opportunity to examine the intellectual neighbourhood from a perspective independent of Western knowledge systems. This effort aims to highlight the interconnectedness of knowledge circulation, a phenomenon that we must acknowledge and appreciate. The first section explores the relationship between Ulugbek, a king and astronomer, and his protégé, Ali Qushji. Ulugbek established a scholarly sanctuary that fostered a lineage of eminent thinkers in Central Asia. Ulugbek and Ali Qushji cultivated a legacy of intellectual camaraderie, the effects of which continue to resonate across Eurasia. The paper's second section examines the transmission and transformation of classical astronomical knowledge in Istanbul. This part focuses on how the works of ancient astronomers like Ptolemy were interpreted and expanded upon in the cosmopolitan environment of Istanbul, significantly influencing the regional approaches to astronomy.

In the third section, ‘The new round of Intellectual Neighbourhood: Ali Qushji and Regiomontanus,’ the focus shifts to the interaction between Eastern and Western astronomical traditions. This section details how Ali Qushji’s works influenced the European astronomer Regiomontanus, highlighting a significant intellectual exchange that bridged cultural and geographic divides in pursuing scientific advancement. The conclusion synthesises the discussions and reflects on the continuous thread of knowledge sharing and collaboration that extends from the Ali Qushji era and land to subsequent generations, underscoring the universal quest for understanding among neighbours.

## The premise

The understanding of the intellectual neighbourhood often remains absent from scientific discourse due to the prevailing dominant knowledge system. As Dipesh Chakrabarty explains, ‘for generations now, philosophers and thinkers shaping the nature of social science […] these statements have been produced in relative, and sometimes absolute, ignorance of the majority of humankind, i.e. those living in non-Western cultures.’ ^8^ Perhaps some of us remember Friedrich Engels, whose evocative portrayal pictured the Renaissance as an era that ‘called for giants and produced giants – giants in power of thought, passion, and character, in universality and learning …. The first decisive advance was made by Copernicus…. from then on, the die was cast: the old view might still drag on for a long time, but it was irrevocably condemned.’ ^9^ Engels’ lens, with its acute focus on the European continent and crystallised through the eminent triad of Copernicus, Galileo, and Newton, reveals a constrained vista that predominantly valorises Western scientific ascendancy. Chakrabarty posits that these narratives revolve around the concept of historical transformation, framing the third world within the confines of history as a mere ‘waiting room, a period which is needed for the transition to capitalism at any particular time and place’. ^10^

This standpoint permeates many structures around, not least the institutions of knowledge production - universities. Across the world, including in Central Asia, higher education follows a Hegelian dialectical analysis, where the development of Eastern and Western identities is perceived in juxtaposition. One can adapt to limitations. The modern matrix of university knowledge is constructed on the so-called ‘subject’ in here and ‘object’ out there. Our university syllabus imitates traditional Eurocentric Cartesian summary when the ‘reflection [that] can only roam in its own specific playing field ( *maydan*)’. Likely, ‘one half of the term [in our case, our wish to make historical base akin to European] is artificially privileged over the other for ultimately ideological purposes’. ^11^ The caution came from Ibn Arabi from Al-Andalus, a historical region known for its significant Sufi contributions to Eurasia. Typically, one side is celebrated for its positive attributes, as Engels celebrated Copernicus, Galileo, and Newton, while the other, perceived as lacking them, is relegated to the margins. This relegation may lead to a crisis of self-denial for those whose contributions are marginalised. In this dichotomy, the absence of a mediating presence to encourage dialogue often elevates one side over the other. The ongoing perpetuation of Eurocentrism within the humanitarian disciplines, especially in intellectual history, is evident. When there is a deficit of professional recognition or resources for studying ‘intellectual neighbourhood’ and translating those neighbours who express their thoughts and ideas in non-European languages, these figures and their ideas will be ignored from the history syllabi. Consequently, the ‘specific playing field’, as noted by Ibn Arabi, which remains deeply rooted in Eurocentric visions, can ultimately appear unpersuasive.

The dialogue transcends the boundaries of Engel’s giants, aligning more broadly with the concepts articulated by Al Ghazali in the twelfth century, encapsulated in the triad of ‘time, place, and fellowship’ ( *zaman, makan, akhwan*). ^12^ Engels emphasises the role of exceptional individuals from a specific geographical background in intellectual development. Al Ghazali offers a broader and more interactive vision of knowledge because knowledge is never an individual project. His perspective calls for a collaborative process in which various voices engage in critical dialogue, cross-disciplinary exchange, and intercultural conversation spanning different eras. Al Ghazali invites to review a context (not a geography) that necessitates the presence of dynamic contributors—those prepared to interrogate, refine, and converse. Within this expansive intellectual fabric, a broad and interwoven neighbourhood of thought flourished, one that extended its reach from the learned circles of Samarkand to the halls of Toledo, spanned from the academies of Beijing to the libraries of Istanbul, and further cast its influence over the intellectual landscapes from Istanbul to the universities of Bologna and Paris. Perhaps through this prism of intellectual neighbourhood, we can overcome Western tradition ‘to tailor issues to manageable, individual or at least sub-disciplinary sizes’ with numerous regulations. ^13^ It is redundant to say, that the urgency of problematising alternatives to the Western ‘tailoring’ is brought upon us by complexities and lack of horizontal intercultural dialogue in Central Asia, as well as other parts of the Globe.

Grasping the development of intellectual traditions, particularly within the realms of scientific and cultural discourse, necessitates acknowledging the indispensable influence of Islamic scholarly tradition. This tradition emerged from exploring the intellectual heritage of their ‘intellectual neighbourhood,’ intersecting with Greek, Indian, Persian, and Syrian philosophies. These philosophical foundations significantly informed Islamic natural philosophy, propelling a robust and wide-ranging interchange of intellectual insights. This process is a beacon of extensive intellectual diversity and solidarity, bridging geographical and cultural divides. Within this framework, the reciprocal influences and resonances between Chinese and Indo-Persian cultures become apparent, alongside the nuanced artistry and symbolism embedded within Persian literary conventions such as ‘Sabqi Hindi’ in India. The contemplation of historical scholastic symbioses further enriches this narrative, with pairs like Ibn Arabi (1165 – 1240) from Al-Andalus and Abdurrahman Jami (1414 – 1492) from Herat or Ibn Sina (980—1037) from Bukhara and his influence on the story of Dante’s Alighieri (1265—1321) ‘Divine Comedy’ serving as prime examples. ^14^

In exploring the dynamic neighbourhood between natural philosophy and theological concerns through the lens of astronomy, the contributions of Nasir ad-Din Tusi (1201–1274) are instrumental. Indeed, Tusi’s scholarly lineage is evident in the groundbreaking contributions of Ulugbek and his student, Ali Qushji. Their work, deeply influenced by Tusi’s intellectual framework, further expanded the boundaries of astronomical science, securing their place as distinguished figures of the intellectual community of neighbours from Eurasia.

## Ulugbek and Ali Qushji: Neighbours in the Pursuit of Astronomical Knowledge

Ulugbek, the grandson of the illustrious Timur (1336–1405), who profoundly impacted the Eurasian continent through his fourteenth-century conquests, chose a path distinct from his ancestor’s martial exploits. Instead of pursuing military glory, Ulugh Beg embraced the synthesis of politics and science, fostering an environment that encouraged the intellectual growth of a community of talented scholars, one of whom was the renowned young astronomer Ali Qushji. Ulugbek’s intellectual and administrative achievements were closely connected to Mawarannahr. He presided over the region for four decades, from 1409 to 1449. In the region under his management were the establishment of the observatory in Samarkand and a series of madrassas, notably in Samarkand (1417-1420), Bukhara (1417), and Gijduvan (1433). In Samarkand, the capital of Timur, the Ulugbek Madrassa was known initially as *Madrasai Oliya.* ^15^ All those institutions distinguished themselves by offering comprehensive education in religious studies and advanced fields such as mathematics and astronomy.

Ulugbek observatory, known as *Rasadkhona* in Tajik-Persian, was established in Samarkand between 1428 and 1429. The observatory featured a cylindrical geometric design, measuring 48 metres in diameter and 35 metres in height, with a radius of 40 metres. It became popularly known as *Nakshi Jahon* (Project of the World). ^16^ The architectural plans, necessary instruments, and types of astronomical observations were discussed during a significant assembly of esteemed intellectuals from various regions of Central Asia at that time. This venture brought forth the monumental Fakhri sextant within the observatory. This tool paid homage to the pioneering work of al Khujandi (who lived in the late 10th century) and his benefactor Fakhr al-Dawla (which is why the sextant was named after Fakhri). It stood as a hallmark of the most refined astronomical exactitude. ^17^ Ulugbek established a rigorous observational schedule for the team of the observatory, mandating the surveillance of the Sun and Moon without fail each day, Mercury every five days, and comprehensive scrutiny of the remaining planets on a ten-day cycle, thus ensuring a meticulous and enduring record of the heavens. ^18^ Among those who participated from the very start was a young Ali Qushji, who was 25 years old when the observatory was constructed. Through this experience, he began to understand the importance of having practical resources and good teachers to work in the observatory effectively. In addition to the observatory, there was a carefully curated library of the father of Ulugbek - Shohruh. Ali Qushji had access there as a royal court member (the son of the king’s falconer). Among prized possessions of the collection were notable works such as ‘Zij-i Ilkhani’ (The Astronomical Tables of Ilkhanids) by Nasir ad-Din al-Tusi, and the astronomical treatise ‘The Book of the Fixed Stars’ (964) [kitāb suwar al-kawākib]. ^19^ The members of the Ulugbek team frequently annotated these texts with their observations, including Ulugbek himself, showcasing his operational engagement with the material.

Amidst the intellectual contributions, the New Astronomical Table emerges as a seminal work, representing a synergy between Ulugbek and Ali Qushji. Known in Tajik-Persian as ‘Zijji Sultani’ and in Turkish as ‘Zij-i Jadidi Kuragoniy,’ ^20^ this star catalogue, completed in 1437, stands as a pinnacle of precision in the field, documenting the coordinates of 1,018 stars with extraordinary accuracy. ^21^ It is one of the best-preserved written sources on medieval astronomy and mathematics before the invention of the telescope, with 108 handwritten copies still in existence. ^22^

This compendium of astronomical knowledge travelled extensively along what some scholars would note as the Silk Road, read and appreciated by learned societies from the Far East to the West. Its reverence was such that it garnered official sanction by the Mamluk leaders (1250-1517) for distributing these essential celestial tables. The Egyptian scholars neatly translated the astronomical data into Arabic, aligning the calculations with the specific skies above Cairo. Over time, these astronomical tables were assimilated into the cultural and scientific practices of the Ottoman, Mughal, and Safavid empires, becoming integral to the intellectual landscapes of major cities like Isfahan, Istanbul, and Delhi. Their extensive use played a crucial role in standardising the observance of Islamic religious rituals and methods of orientation and travel throughout vast regions, including areas beyond Dar al-Islam. In 1648, John Greaves (1602 – 1652), a professor of astronomy at the University of Oxford, partially published this work for the first time in Oxford. ^23^ The most detailed analysis in English was published in the USA in 1917 by E. B. Noble under the title ‘The Star Catalogue of Ulugh Beg: Revised after all Persian Manuscripts Existing in Great Britain’. ^24^ This catalogue served as a beacon of astronomical knowledge and as a demonstration of the Distant Intellectual Neighbourhood, showcasing its profound impact that reached well beyond its initial Timurid sphere.

In this vein, Ali Qushji further accelerated the scholarly dialogue by engaging critically with Tusi’s ‘Tajrid al-i’tiqad.’ Notably, while Tusi challenged specific geometrical aspects, he did not extend his critique to the fundamental Ptolemaic geocentric paradigm. However, Ulugbek and Ali Qushji boldly questioned the prevailing astronomical paradigms, advocating considering Earth’s rotation as a plausible hypothesis. ^25^ This move deliberately stepped away from the confines of Aristotelian physics.

Ali Qushji’s scholarly work ‘Risala fi hall ishkal al-mu addil li-l-maslr,’ commonly known as the Mercury treatise, analysed the motions of the planet Mercury. ^26^ The young researcher proposed a new model to explain its observed irregularities. Ali Qushji offered a novel solution to the longstanding Equant Problem within astronomical models, explicitly focusing on Mercury. The treatise, composed before Ulugbek’s premature death in 1449, is an enduring testament to a time when the ‘king and astronomer’s’ mentorship and intellectual influence significantly impacted Ali Qushji’s scholarly work. Furthermore, the text highlights Ulugbek’s scholarly dedication to consistent celestial motion, decisively distancing from Ptolemy’s equant point methodology. This argument opened new avenues for the accepting and exploring diverse astronomical theories, marking a transformative moment favouring empirical inquiry over established philosophical assertions in the quest to comprehend the universe. The Ulugbek team, with Ali Qushji as a notable member, diverged from Ptolemy’s approach. Instead, they adhered to the mediaeval astronomical principle that celestial bodies should rotate uniformly around their actual centres, rejecting the theoretical equant points of Ptolemy’s model. ^27^ In adopting this stance, they positioned themselves among the learned elite who questioned prevailing astronomical paradigms, particularly the non-uniform rotational patterns foundational to Ptolemy’s cosmic schematics.

## Cosmopolitan Istanbul and the Legacy of Ptolemy’s ‘Almagest’

The fortunes of Samarkand shifted dramatically, bringing the epoch under Ulugbek to a close in the year 1449. Ali Qushji embarked on a long-lasting quest to find new sanctuaries where his academic pursuits could flourish anew. In 1472, he arrived in the city, which, after the Muslim invasion in 1453, began to be referred to as Istanbul (from the Greek phrase ‘eis tin Polin’, meaning ‘to the city’). The transformation of the city’s name symbolises not only a change in the political regime but also the emergence of a new intellectual setting, mirroring the scale of the Eurasian intellectual neighbourhood. It is clear that ‘to override the paradox and create connectivity story we would need the city that mystically intertwined hybrid identities and acts to centre the margins.’ ^28^ In the case of cosmopolitan cities, such as Samarkand and Istanbul, hybrid identity focuses less on the narrow national (including linguistic) priorities and more on the intellectual neighbourhood. It emerges from the environment of those cities, which stimulates and is conducive to philosophical discourses.

One more meaningful comparison is that two key historical figures, Mehmed II and Timur - two conquerors of Eurasia ‘though separated by time and geography, shared a vision of an empire that combined military might with cultural patronage. Mehmed’s transformation of Constantinople into a vibrant capital mirrors Timur’s efforts to elevate Samarkand as a cultural hub, showcasing how both leaders understood the importance of culture in legitimising their rule.’ ^29^ In Istanbul, akin to Samarkand of Timurid time, government functionaries proficient in mathematics and surveying were at the forefront of the various efforts. They aimed to enhance their expertise and, consequently, their administrative power. In this environment, knowledge transcended academic confines and became integral to political strategy, merging scholarly aptitude with the gears of statecraft. Researchers have identified a dichotomy within this learned society: some traditionalists followed the age-old Ptolemaic system, which had long been the foundation of celestial studies.

In contrast, a progressive faction emerged, advocating for reliance on Islamic astronomical treatises, as well as the rich heritage found in the numerous manuscripts from all over Dar al Islam. Some of them incorporated regional observations and innovations that diverged from Ptolemy’s geocentric model, suggesting a vibrant intellectual debate and a diverse approach to understanding the cosmos within the astronomical community. When Ali Qushji arrived in Istanbul, the dynamic of ‘time, place, and scholarly fraternity’ (Al Ghazali's perception) created a fertile ground for intellectual contest, enhancement, and exchanges. Renowned for the membership of the legendary Samarkand’s team of astronomers and his scholarly achievements, Ali Qushji drew the interest of Sultan Mehmed II promptly upon his arrival. It led to the appointment of Ali Qushji as a mathematics professor at the revered Aya Sofia.

Although Ali Qushji’s tenure in Istanbul was brief, lasting a mere two years, his noteworthy contributions to astronomy and mathematics captured the attention of learned circles of the city and beyond. He presented several treatises which were concluded or re-evaluated during his stay. Among them, the most perhaps significant one was the Mercury treatise, titled ‘Risala fi hall ishkal al-mu’addil li-l-masir’, in which he addressed and discussed the complex Equant Problem. As mentioned, the treatise was initiated in a collaborative effort guided by Ulugbek, whose contributions were integral and widely acknowledged within the text. ^30^ The work represents a significant advancement of earlier texts by Al-Tusi, notably the Zij-i Ilkhani and Tejrid al-Kalam (Abstract of Theology). ^31^

Reflecting on the ongoing discourse surrounding Ptolemy’s seminal work in the scholarly circles of Tusi, Ulugbek, and Ali Qushji, it’s crucial to appreciate the enduring legacy of Ptolemy’s ‘Almagest.’ ^32^ Crafted in the 2nd century by Claudius Ptolemy, this extensive text on the dynamics of celestial phenomena has been pivotal in shaping the astronomical narrative over millennia. It glorified the geocentric perspective, a dominant scientific doctrine that emerged from the scholarly melting pot of Hellenistic Alexandria and resonated through successive cultures and epochs, influencing Islamic and European thought. Recognition of specific academic works is essential because they foster intellectual discourses among diverse cultural realms. In this milieu, the work of Basilios Bessarion (1403-1472), a Roman Catholic cardinal-bishop and a distinguished scholar of Greek, stands out as particularly significant. ^33^

Bessarion was a crucial figure in the enlargement of fifteenth-century European astronomical knowledge. His profound influence is especially evident in how he facilitated the spread and explication of the Almagest, enriching it with the considerable contributions and offerings from Islamic astronomy. It is important to remember that while Tusi took issue with specific geometric components, he accepted the essential Ptolemaic geocentric model. The challenge to this paradigm came through the works of Ali Qushji, particularly regarding Mercury (mentioned ‘Risala fi hall ishkal al-mu’addil li-l-masir’). Bessarion’s impressive collection of texts and manuscripts, sourced from Constantinople and other places, included works of Nasir al-Din Tusi, his famous ‘Ziji Ilkhani.’ In that manuscript, Tusi referred to it alternately as ‘The Paradosis of the Persian Tables’, a source on astronomy between the Ilkhanate and the Eastern Roman Empire. ^34^ Furthermore, Bessarion’s collection of Persian works, preserved in various manuscripts like ‘Marcianus graecus Z.323, Marc.gr.328, Marc.gr. Z.336, and Marc. lat. VIII 31’, ^35^ is of immense scholarly value—the Marc.gr. Z323 manuscript, a fourteenth to fifteenth-century codex, is particularly notable for its inclusion of Ptolemaic and Islamic astronomical works and Greek mathematical and geometric texts, showcasing the extensive and diverse scholarly materials at Bessarion’s disposal.

Significantly, through the adept translations into Greek by scholars such as Bessarion, a substantial body of Islamic astronomical treatises was incorporated into Greek scholarship. ^36^ His disciples gleaned from the study of these texts, especially from the writings of Tusi, a profound insight: a point’s linear reciprocating motion could be accounted for by the orbital rotations in space in the context of Ptolemaic astronomy. This mechanism, known as the ‘Tusi couple’, was significant because it provided a geometrical solution to the question of uniform circular motion, which was a task in the Ptolemaic system that posited that planets moved in perfect circles. ^37^ A small circle rotates within a larger circle that has twice the diameter. As this occurs, a point on the perimeter of the larger circle exhibits a straight-line motion. This results from the combined uniform circular movements of the two circles.

Tusi and Al Qushji’s interpretation of Ptolemaic heritage illustrates the groundbreaking character of Islamic scholars during that period. Their works, along with those from various regions of the Dar al-Islam, such as Persia and Central Asia, were brought to Europe through the efforts of scholars like Bessarion. This cross-cultural exchange highlights the interconnectedness of scientific thought across cultures and periods.

### The new round of Intellectual Neighbourhood: Ali Qushji and Regiomontanus

Engel’s idea that ‘the first decisive advance was made by Copernicus …’ continues to influence many universities worldwide. However, we have an opportunity to examine the Copernican revolution from a different perspective through Al Ghazali’s vision. In that case, history became much more interconnected through neighbours. Before Engel’s ‘the first’, we had Bessarion’s student, Regiomontanus (1436 – 1476). He was a critical figure who brought complex ideas and interpretations of his teacher, Bessarion, to the forefront of European scholarly circles. Having encountered him in Vienna and mastered Greek under his influence, Regiomontanus translated the Greek manuscript of the Almagest into Latin. With the great assortment of ideas from previous intellectuals, this critical work set the stage for his later fundamental work, the ‘Epytoma in Almagestum Ptolemaei’, which was published in 1496 and marked a significant progression in astronomical scholarship. Regiomontanus’s translation was more than a mere transmission; it enhanced the original treatise with a nuanced comprehension of the inner planetary movements, thus deepening astronomical insights for his contemporaries and successors, among whom we typically remember Copernicus.

We must pay particular attention to an essential element in this discourse: examining Book XII, Chapters 1 and 2 of Regiomontanus’s Epitome of the Almagest reveals intriguing connections, illuminating the sustained intellectual Neighbourhood. Jamil Ragep, a prominent scholar from McGill University, highlighted, ‘Recently it has come to light that the critical proposition Copernicus used to transform the epicyclic models of Mercury and Venus into eccentric models, which is found in Regiomontanus’s Epitome, was put forth earlier in the fifteenth century by ʿAlī Qushjī of Samarqand.’ ^38^ It illustrates not only the transfer of knowledge across cultures and eras but also the foundational role of these earlier scholars in setting the stage for the revolutionary cosmological shifts of the Renaissance. This insight affirms the depth of the intellectual debt that early modern European astronomy owes to its predecessors, bridging the gap between medieval Islamic astronomy and the dawn of the Copernican revolution.

## Conclusion

The surge in astronomical learning in Europe did not emerge in a vacuum. It is accurate for Poskett J. to assert the following: ‘Copernicus relied on mathematical techniques borrowed from Arabic and Persian texts. When Newton set out the laws of motion, he relied on astronomical observations from Asia and Africa. Darwin consulted a sixteenth-century Chinese encyclopaedia when writing On the Origin of Species. And when Einstein was studying quantum mechanics, he was inspired by the Bengali physicist, Satyendra Nath Bose.’ ^39^ In all these instances, we discuss a vital intellectual neighbouring network that has been and continues to be crucial in the Eurasian context of both the past and present.

The story is a testament to the collaborative spirit of Al Ghazali inquiry. The vibrant intellectual neighbourhood, fostered by scholars from diverse cultural backgrounds, still echoes through time, underscoring that knowledge creation is intrinsically a collective intercultural enterprise. As contemporary global crises emerge, acknowledging and valuing this interconnectedness becomes fundamental. Embracing such connections is essential for cultivating understanding and respect toward each other and advancing a wide-ranging appreciation of our neighbouring intellectual legacy.

## Data Availability

No data are associated with this article.

## Footnotes

^1^

*International Alī al-Qūshjī Symposium* (Istanbul, November 18–20, 2024), Istanbul Medeniyet University, Institute of History of Science, and Turkish Academy of Sciences (TÜBA); *International Conference: Channels of Transmission of Astronomical Knowledge in the Ottoman World (14th–18th Centuries)* (Istanbul, November 21–24, 2023), Istanbul University, Department of the History of Science, and Institut Français d’Etudes Anatoliennes.

^2^
“Nasir al-Din Ṭūsī,” *Encyclopædia Iranica*, accessed March 25, 2024, https://www.iranicaonline.org/articles/tusi-nasir-al-din-bio.

^3^
Tashmukhamed Niyazovich Kary-Niyazov, *Selected Works: In 8 Volumes. Astronomical School of Ulugbek*, vol. 3 (Tashkent: Fan, 1967), 374.

^4^
Ali Qushji, *Arithmetic Treatise*, trans. U. Attayev (Samarkand: SamGU, 1972).

^5^
Claudius Ptolemy, *Almagest*, trans. G. J. Toomer (London: Duckworth, 1984).

^6^
Alberto Bardi, “Islamic Astronomy in Fifteenth-Century Christian Environments: Cardinal Bessarion and His Library,” *Journal of Islamic Studies* 30, no. 3 (September 2019): 338–66. DOI: 10.1093/jis/etz013.

^7^
Johannes Regiomontanus, *Epytoma in Almagestum Ptolemaei* (Venice, 1496), described by Sophia Rare Books, accessed September 4, 2024, https://www.sophiararebooks.com/pages/books/5855/ptolemy-johannes-regiomontanus-johannes-muller-and-georg-peurbach/epytoma-in-almagestum-ptolemaei .

^8^
Dipesh Chakrabarty, *Provincializing Europe: Postcolonial Thought and Historical Difference* (Princeton: Princeton University Press, 2000), 29.

^9^
Friedrich Engels, *Dialectics of Nature* (Moscow: Marxist Internet Archive, 1925), accessed June 10, 2014, https://www.marxists.org/archive/marx/works/1883/don/index.htm.

^10^
Chakrabarty, *Provincializing Europe*, 65.

^11^
Ian Almond, *Sufism and Deconstruction: A Comparative Study of Derrida and Ibn ʿArabi* (New York: Routledge, 2004), 24.

^12^
Nargis T. Nurulla-Khoja, “Decolonisation via New Understanding of Sufism,” *MGIMO Review of International Relations* 60 (2018): 139–60. DOI: 10.24833/2071-8160-2018-3-60-139-160.

^13^
“Guest Editors’ Introduction: Philosophy and Its Borders,” *Dialogue: Canadian Philosophical Review* 59, no. 1 (2020): 1–3. DOI: 10.1017/S0012217320000023.

^14^
Munira Shahidi, *Ibn Sino i Dante* (Dushanbe: Donish, 1985), 127; Munira Shahidi, “‘Abu Ali Ibn Sina obitatel Limba,’” in *Dantovskie chteniya* (Moscow, 1985), 151–74.

^15^
Abdusattor Jumonazar, *Bukhara: The History of the Education System* (Tashkent: Akademnashr, 2017), 68.

^16^
Ibid.

^17^
M. S. Bulatov, “Ulugbek Observatory in Samarkand,” *Historical and Mathematical Studies* 18 (1986): 199–216.

^18^
Ibid., 203.

^19^
Ibid., 206.

^20^
E. S. Kennedy, *A Survey of Islamic Astronomical Tables*, *Transactions of the American Philosophical Society* 46, no. 2 (1956): 3–4, 44–45.

^21^
Tashmukhamed Niyazovich Niyazov, *Astronomical School of Ulugbek, Selected Works*, Vol. 6 (1967).

^22^
B. Pasilov, *Ulugbek* (Tashkent: Аbu Matbuot-Konsalt, 2011), 23.

^23^
John Greaves, *Binae Tabulae Geographicae* (London, 1648).

^24^
E. B. Knobel, *Ulugh Beg’s Catalogue of Stars* (Washington: Carnegie Institution of Washington, 1917).

^25^
Kary-Niyazov, *Selected Works*, 87.

^26^
George Saliba, “Al-Qushjī’s Reform of the Ptolemaic Model for Mercury,” *Arabic Sciences and Philosophy* 3 (1993): 161–203.

^27^
Ibid., 169.

^28^
M. A. Al-Azmeh, “The City as a Site of Hybrid Identities,” in *The City in the Islamic World*, ed. Michael Dumper and Bruce E. Stanley (Brill, 2018), 1–20.

^29^
Caroline Finkel, *Osman’s Dream: The History of the Ottoman Empire* (New York: Basic Books, 2005), 37.

^30^
Saliba, “Al-Qushjī’s Reform of the Ptolemaic Model for Mercury,” 179.

^31^
“Ali Kuşçu,” Ahya.net, accessed June 1, 2024, https://ottoman.ahya.net/konu-2090-z18.html.

^32^
J. P. Britton, *Models and Precision: The Quality of Ptolemy’s Observations and Parameters* (New York: Garland, 1992).

^33^
N. G. Wilson, *From Byzantium to Italy: Greek Studies in the Italian Renaissance* (London: Duckworth, 1992).

^34^
Alberto Bardi, “The Paradosis of the Persian Tables,” *Journal for the History of Astronomy* 49, no. 2 (2018).

^35^
Ibid.

^36^
David Pingree, *The Astronomical Works of Gregory Chioniades*, vol. 1 (Amsterdam: J.C. Grieben, 1985), 18.

^37^
Michela Di Bono, “Copernicus, Amico, Fracastoro and Ṭūsī’s Device,” *Journal for the History of Astronomy* 26, no. 2 (1995): 133–54.

^38^
F. Jamil Ragep, “Alī Qushjī and Regiomontanus,” *Journal for the History of Astronomy* 36, no. 4 (2005): 359–71.

^39^
James Poskett, *Horizons: A Global History of Science* (New York: Viking, 2022), 1-2.
