# Supplementary material for: Reconstructing the intellectual neighbourhood: tracing Ali Qushji’s influence in science
Source: F1000Res. 2025 Mar 13;14:288. [Version 1] doi: 10.12688/f1000research.162047.1 (PMC11992511; doi:10.12688/f1000research.162047.1)
Supplement: Peer review history

# Reviewer report: Reviewer response for version 1

**DOI:** 10.5256/f1000research.178172.r374070

**Published:** 2025-06-20

**Authors:** Encep Abdul Rojak

**Affiliations:** 1 UNIVERSITAS BANDUNG, Bandung, Indonesia

This research is fascinating to explore further in the field of astronomy. Moreover, this figure collaborated with others in compiling astronomical tables, such as the *Zij-i Sultani* table alongside Ulugh Bek. The data produced by the astronomer Ulugh Bek is well-known and has even reached Indonesia as one of the references revitalizing Islamic astronomical data in the country. However, this study does not explain the research steps carried out, including the development of the problem background, primary data collection, data collection methods, and the analysis used.

Is the review written in accessible language?

Yes

Are all factual statements correct and adequately supported by citations?

Yes

Are the conclusions drawn appropriate in the context of the current research literature?

Yes

Is the topic of the review discussed comprehensively in the context of the current literature?

Yes

Reviewer Expertise:

It is necessary to explain the research steps taken, not merely mentioning the historical sources but also including the steps required to uncover those historical data.

I confirm that I have read this submission and believe that I have an appropriate level of expertise to confirm that it is of an acceptable scientific standard.

# Reviewer report: Reviewer response for version 1

**DOI:** 10.5256/f1000research.178172.r374077

**Published:** 2025-04-11

**Authors:** Mahmoud Ahmed

**Affiliations:** 1 Cairo University, Giza, Giza Governorate, Egypt

Dr Nurulla-Khoja’s article presents a thoughtful and well-researched intervention into the history of science through the concept of the “intellectual neighbourhood”, a framework that foregrounds the cross-regional and dialogic conditions under which knowledge was produced in the late medieval and early modern Islamic world. By tracing the scholarly continuum from Tusi to Ulugbek and Ali Qushji, and further to Regiomontanus and Copernicus, the author illustrates how mathematical astronomy circulated through carefully maintained scholarly and institutional infrastructures across Eurasia. The work makes strong use of primary sources in Persian, Arabic, and Latin traditions and offers detailed accounts of observatory practices, manuscript transmission, and theoretical critique, particularly in relation to the equant problem.

 One of the article’s key strengths is its grounding in material and spatial realities: Samarkand’s observatory, Istanbul’s scholarly circles, and the trans-imperial journeys of texts like the *Zij-i Sultani*. The framing of cities as sites of knowledge encounter, where hybrid practices emerge through scholars’ movement, instruments, and ideas, is especially compelling. The article’s treatment of the relationship between astronomy, empire, and education is careful, and helps shift the narrative away from isolated genius models toward a more distributed model of inquiry. However, the piece might have further developed how these intellectual currents were embedded within broader social or ritual contexts: for example, how astronomical knowledge intersected with calendrical governance, imperial legitimation, or ceremonial life, especially in the Ottoman or Mughal settings.

 The article is clear, accessible, and refreshingly transregional in scope, with potential relevance well beyond the history of science. The “intellectual neighbourhood” is introduced not just as an analytical device but as a historiographical challenge to inherited disciplinary divisions. The concept is promising, and it would be compelling to see a more direct discussion of its applicability beyond astronomy; for instance, to architectural planning, artistic exchange, or diplomatic practice. Additionally, a clearer articulation of the methodological implications (how one might operationalise “intellectual neighbourhood” in future research?) would extend its utility for scholars across fields. Further elaboration on the role of translation as both epistemic and political work could also strengthen the argument, given its centrality to the cross-cultural flows the article traces. I believe the article is a valuable contribution to ongoing efforts to reconstruct horizontal histories of knowledge and to reframe Eurasian intellectual heritage on its own terms. I support its indexing. 

 Refer [1]

Is the review written in accessible language?

Partly

Are all factual statements correct and adequately supported by citations?

Yes

Are the conclusions drawn appropriate in the context of the current research literature?

Yes

Is the topic of the review discussed comprehensively in the context of the current literature?

Yes

Reviewer Expertise:

medieval central Asian history, history of Islamic architecture, central Asian civilization

I confirm that I have read this submission and believe that I have an appropriate level of expertise to confirm that it is of an acceptable scientific standard.

## References

[1] : Encyclopedia of the History of Arabic Science.2019; 10.4324/9780203403600 10.4324/9780203403600

# Reviewer report: Reviewer response for version 1

**DOI:** 10.5256/f1000research.178172.r374069

**Published:** 2025-04-04

**Authors:** Munira Shahidi

**Affiliations:** 1 University of Cambridge, Cambridge, UK

Writing as a scholar of Central Asia whose work is shaped by the intellectual heritage of Samarkand, Bukhara, and Herat, and who has long been attentive to the transmission of thought across literature, languages, and philosophical traditions, I found this article both illuminating and intellectually strong. It speaks not only to a lineage of astronomers and philosophers, but to a particular ethical and epistemic horizon - one in which knowledge is nurtured through trust, reciprocal inquiry, and the carefully cultivated proximities of neighbours across time and space.

 This article offers a layered contribution to the global history of science and to intellectual history more broadly. By tracing the legacy of Ali Qushji within an expansive Eurasian constellation, the author introduces the concept of the 'intellectual neighbourhood' - a methodological framework that foregrounds the collaborative, cross-cultural, and inherently relational nature of knowledge-making. The term is not proposed metaphorically, but as a serious philosophical intervention, grounded in the conviction that 'knowledge is never an individual project', and that it flourishes through contexts that 'foster compromises in trade, science and diplomacy, allowing for the adaptation of local contexts to incorporate the influences of others'.

 The piece takes the reader across philosophical, cosmological, and historical registers, weaving together a narrative that is at once empirically grounded and conceptually ambitious. Especially striking is the invocation of Al Ghazali’s triad of *zaman*, *makan*, and *akhwan*, which is not deployed ornamentally, but functions as a kind of counterpoint to Cartesian and Eurocentric paradigms of linear development. The author does not seek to simply provincialise Europe, but rather to restore a sense of horizontal intimacy to the circulation of thought - one where 'dialogue transcends the boundaries of Engels’ giants', and where the intellectual fellowship of cities such as Samarkand and Istanbul emerges as central to the global movement of ideas.

 Ali Qushji is positioned not merely as an isolated figure of brilliance, but as part of an evolving network of inquiry that includes the likes of Nasir al-Din Tusi, Ulugbek, and Regiomontanus. The discussion of *Risala fi hall ishkal al-mu addil li-l-masir*, and the re-evaluation of the equant problem, is handled with clarity and insight. The author’s reading of the *Zij-i Jadidi Kuragoniy*, and its circulation 'from the Far East to the West', further anchors the argument in material histories of transmission. It's this rethinking of scientific method that also is particularly befitting for F1000Research journal. The article’s insistence that cities such as Istanbul became 'bridges between neighbours', in a way we don't often think about, is especially welcome. In this respect, the article sits comfortably alongside recent work in connected and entangled histories, while retaining a distinctive voice that draws from Central Asian epistemic idioms. The article prompted reflection on how existing scholarship - such as El-Rouayheb’s mapping of Ottoman-era intellectual currents or Ahmed’s challenge to fixed definitions of Islamic thought - might be reread through the lens of neighbourhood, not only as a spatial metaphor but as an ethical architecture of knowledge.

 Factual statements are consistently well-supported. The references span an impressive range of traditions and languages, with commendable attention given to, often dismissed, texts in Tajik-Persian. That the author includes manuscripts such as the *Marc.gr. Z323*, or reflects on John Greaves’ 1648 publication of the star tables in Oxford, speaks to a method that is archival as well as philosophical. The engagement with canonical sources (Ptolemy, Tusi) is balanced by a willingness to cite lesser-known yet deeply significant materials, such as the works of Basilios Bessarion and his 'Paradosis of the Persian Tables'.

 The writing is accessible and attentive. It offers moments of quiet beauty without excess - phrases such as 'the fertile distances between neighbours' or 'a virtual museum of mobility' stay with the reader, not for their flourish but for the precision with which they name difficult concepts. The metaphors do not obscure the analysis; rather, they draw the reader more deeply into it.

 One gentle suggestion would be to reflect slightly more on how the framework of the past might serve not only as critique but as a springboard for imagining new scholarly futures, though this may well be the subject of the author’s next piece.

 This was a thoroughly enjoyable read, and an excellent piece for F1000Research: erudite, expansive, and refreshing. It offers a genuine methodological alternative to prevailing historiographies of science and introduces a language of scholarly intimacy that feels both historically grounded and urgently contemporary. I recommend it for indexing

Is the review written in accessible language?

Yes

Are all factual statements correct and adequately supported by citations?

Yes

Are the conclusions drawn appropriate in the context of the current research literature?

Yes

Is the topic of the review discussed comprehensively in the context of the current literature?

Yes

Reviewer Expertise:

History of Science; Islamic Intellectual History; Central Asian Studies; Transimperial History; Philosophy of History

I confirm that I have read this submission and believe that I have an appropriate level of expertise to confirm that it is of an acceptable scientific standard.

## References

[1] : Islamic Intellectual History in the Seventeenth Century.2015; 10.1017/CBO9781107337657 10.1017/CBO9781107337657

[2] : What Is Islam?.2018; 10.1515/9781400873586 10.1515/9781400873586
